# Proteomics Applications in *Toxoplasma gondii*: Unveiling the Host–Parasite Interactions and Therapeutic Target Discovery

**DOI:** 10.3390/pathogens13010033

**Published:** 2023-12-29

**Authors:** Bin Deng, Laura Vanagas, Andres M. Alonso, Sergio O. Angel

**Affiliations:** 1 Department of Biology and VBRN Proteomics Facility, University of Vermont, Burlington, VT 05405, USA; 2 Laboratorio de Parasitología Molecular, Instituto Tecnológico de Chascomús (CONICET-UNSAM), Chascomús 7130, Provincia de Buenos Aires, Argentina; vanagas@intech.gov.ar (L.V.); sangel@intech.gov.ar (S.O.A.); amalonso@intech.gov.ar (A.M.A.); 3 Escuela de Bio y Nanotecnologías (UNSAM), 25 de Mayo y Francia. C.P., San Martín 1650, Provincia de Buenos Aires, Argentina

**Keywords:** *Toxoplasma gondii*, proteomics, host–parasite interactions, PTMs, drug discovery

## Abstract

*Toxoplasma gondii*, a protozoan parasite with the ability to infect various warm-blooded vertebrates, including humans, is the causative agent of toxoplasmosis. This infection poses significant risks, leading to severe complications in immunocompromised individuals and potentially affecting the fetus through congenital transmission. A comprehensive understanding of the intricate molecular interactions between *T. gondii* and its host is pivotal for the development of effective therapeutic strategies. This review emphasizes the crucial role of proteomics in *T. gondii* research, with a specific focus on host–parasite interactions, post-translational modifications (PTMs), PTM crosstalk, and ongoing efforts in drug discovery. Additionally, we provide an overview of recent advancements in proteomics techniques, encompassing interactome sample preparation methods such as BioID (BirA*-mediated proximity-dependent biotin identification), APEX (ascorbate peroxidase-mediated proximity labeling), and Y2H (yeast two hybrid), as well as various proteomics approaches, including single-cell analysis, DIA (data-independent acquisition), targeted, top-down, and plasma proteomics. Furthermore, we discuss bioinformatics and the integration of proteomics with other omics technologies, highlighting its potential in unraveling the intricate mechanisms of *T. gondii* pathogenesis and identifying novel therapeutic targets.

## 1. Introduction

*T. gondii* is the causative agent of toxoplasmosis, one of the most common parasitic infections worldwide. The World Health Organization (WHO) estimates that about one-third of the world’s population has been infected with *T. gondii*. Among them, over 800,000 persons have been exposed and an estimated 750 deaths are caused by toxoplasmosis in the United States each year [1].

*T. gondii* engages in both sexual and asexual reproduction, utilizing felines as definitive hosts and other animals as intermediate hosts. Intermediate hosts become infected through consuming oocysts or tissues containing *T. gondii* cysts. Within the intermediate host, the parasite progresses through tachyzoites and bradyzoites development stages. Whereas in the definitive feline host, the sexual stages develop, leading to the excretion of unsporulated oocysts into the environment where they undergo sporulation to become infective, facilitating survival and dissemination.

*T. gondii* employs a range of mechanisms to invade, replicate, manipulate host metabolism, and establish virulence. It invades host cells through a process called gliding motility, which involves the coordinated action of proteins like myosin A, actin filaments, gliding-associated proteins (GAP), microneme proteins, rhoptry neck proteins (RONs), apical membrane antigen 1 (AMA1), and rhoptry proteins (ROPs). These proteins work together to facilitate attachment to host cells, form a moving junction, and, ultimately, the invasion and egress of host cells [2]. *T. gondii* also exhibits varying levels of virulence, with different strains having distinct characteristics. One of the virulence factors is ROP18, which is critical for the parasite’s ability to evade the host’s immune response [3,4]. Virulent strains can cause severe disease in immunocompromised individuals, while less virulent strains may result in milder symptoms or even asymptomatic infections in healthy hosts. Once inside host cells, *T. gondii* replicates quickly and divides within a host cell to form daughter parasites, which are then released to infect new cells. By manipulating the host’s metabolic pathways, *T. gondii* can downregulate the host’s metabolism of fatty acids, lipids, and energy, often through hijacking host-signaling pathways such as peroxisome proliferator-activated receptors (PPAR) [5]. Additionally, the parasite can influence the host’s xenobiotic metabolism, affecting the breakdown of various substances. Understanding these metabolic alterations is crucial for developing targeted therapies.

Clinical outcomes of *T. gondii* infection vary based on the host’s immunity. Immunocompetent individuals typically experience mild symptoms or remain asymptomatic, while immunocompromised cases, like those with HIV/AIDS or organ transplants, face severe complications—encephalitis, retinochoroiditis, and organ damage. Congenital infection can affect fetuses. Existing clinical treatments are limited in scope so far.

Mass spectrometry (MS)-based proteomics, which involves the large-scale study of proteins and their functions, has become a cornerstone of biological and medical research [6,7,8,9,10,11]. Through investigating the dynamic changes in protein expression, PTMs, and protein-protein interactions (PPIs) that occur during *T. gondii* infection, host immune responses, and disease progression, scientists have acquired valuable insights into its biology, host interactions, virulence factors, and potential therapeutic targets [12,13,14,15,16,17,18].

**Figure 1:**
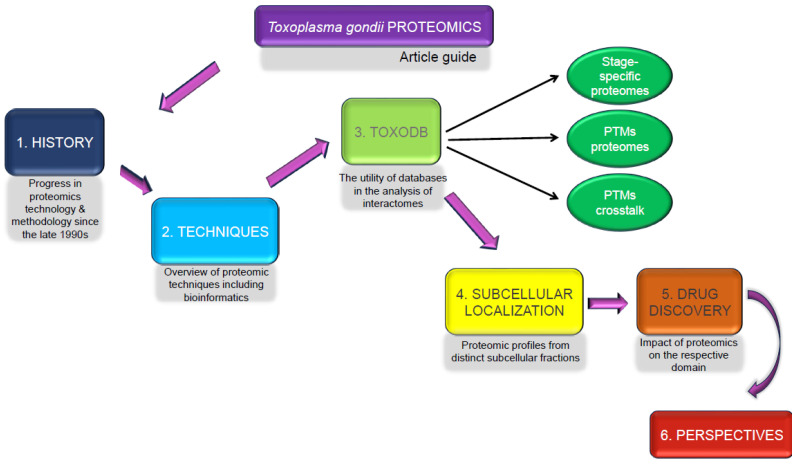
Article overview.

After many years of progress, proteomics has advanced to the point where it can identify and measure around 10,000 proteins from human tissues simultaneously in an experiment [19]. It has played a crucial role in identifying potential biomarkers for diseases caused by parasites and therapeutic targets for treatments. This review aims to spotlight proteomic methodologies applied within these areas and summarize the important findings about key proteins (Table S1) engaged in *T. gondii* invasion and the interactions between the host and the parasite. To enhance the understanding of *T. gondii* biology, we also carried out an in-depth analysis of the proteomes, including stage-specific, PTMs, PTM crosstalk, and subcellular proteome data from the ToxoDB database (Figure 1).

## 2. Proteomics Approaches in *T. gondii* Research

**Figure 2:**
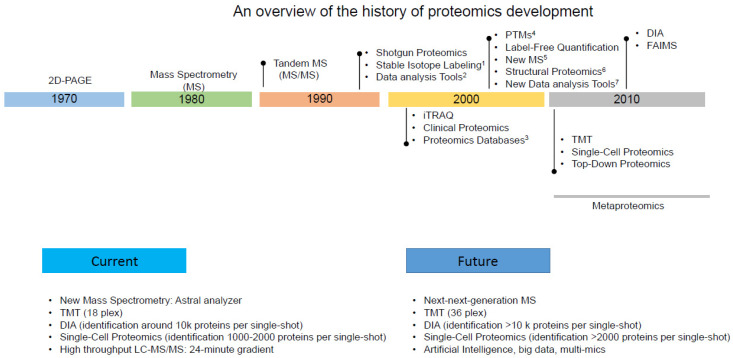
Timeline of the main events associated with proteomic studies. The vertical lines indicate early, middle, or late events depending on their position. Examples of some events: (1) stable isotope labeling by amino acids (SILAC), Isotope-coded Affinity Tag (ICAT); (2) SEQUEST, Mascot, and Scaffold; (3) UniProt, National Institutes of Health database; (4) Phosphoproteome, Acetylome, Ubiqutinome, etc.; (5) Orbitrap, Q-TOF; (6) Hydrogen Deuterium Exchange (HDX)-MS; (7) Skyline, MaxQuant. iTRAQ: Isobaric Tags for Relative and Absolute Quantitation. TMT: Tandem Mass Tags. FAIMS: Field Asymmetric Ion Mobility Spectrometry. DIA: Data-independent acquisition. The decades are not in scale.

Proteomics has evolved significantly over the past few decades (Figure 2) with the advancements in technology and methodology, playing a crucial role in our understanding of the parasite’s biology, host–parasite interactions, and potential drug targets. Here, we will outline the progression of proteomic analyses in general and explore their application in analogous studies related to *T. gondii*. This will provide a comprehensive overview of the rapid advancements in proteomics over the years and its either extensive uses or potential applications in the field of toxoplasmosis research.

### 2.1. Early Studies (Late 1990s to Early 2000s)

Proteomics studies on *T. gondii* began in the late 1990s and early 2000s, primarily using two-dimensional gel electrophoresis (2DGE) combined with matrix-assisted laser desorption/ionization (MALDI) and electrospray ionization (ESI) to separate and analyze proteins. These early studies helped identify and characterize some of the parasite’s proteins [20,21], enabling researchers to analyze thousands of proteins from a biological sample. However, they had limitations concerning sensitivity and the ability to analyze low-abundance proteins.

Shotgun proteomics, also known as bottom-up proteomics, gained popularity in the early 2000s. It involved digesting proteins into peptides and then analyzing those peptides using MS. Several databases, such as UniProt, and searching algorithms, like SEQUEST and Mascot, were established to annotate and search protein sequences and information. These resources have become essential for proteomics research. Liquid chromatography-tandem MS (LC-MS/MS) techniques began to gain prominence in *T. gondii* research due to their improved accuracy in protein identification and quantification [22]. During this stage, researchers could identify a few hundred to a few thousand proteins in a single experiment using techniques like LC-MS/MS coupled with 2DGE. However, this process often required multiple injections of LC-MS/MS, contributing to the overall cost and time investment associated with the research endeavor.

### 2.2. Mid-2000s to the Present

Several key developments and technologies have been established and applied to *T. gondii* research, including the following.

#### 2.2.1. Comparative Proteomics

Comparative proteomics involves comparing the proteomes of *T. gondii* and its infected host cells/tissues under different conditions to identify differentially expressed proteins and reveal molecular changes associated with infection. This approach allows researchers to identify and quantify parasite-specific proteins, host factors targeted by the parasite, and alterations in host protein expression induced by *T. gondii* infection [23,24,25,26,27,28,29]. During this stage, more than 6000 proteins can be identified and quantified using iTRAQ-labeling and strong cation exchange (SCX) fractionation techniques [16].

#### 2.2.2. Subcellular Proteomics

Subcellular proteomics focuses on studying the proteome of specific cellular compartments or organelles involved in host–parasite interactions. By isolating and analyzing proteins from particular subcellular fractions, researchers can identify organelle-specific proteins and elucidate the role of specific compartments in *T. gondii* infection and host response [30,31,32]. Extensive proteomic studies on *T. gondii* have explored subcellular protein localization through diverse analyses, including membrane-soluble fraction comparisons, organelle exploration (e.g., mitochondria), secretory pathway investigation, microvesicle formation, and comprehensive analyses. This research is vital for identifying novel components in various organelles, enhancing our understanding of *T. gondii*’s biological and metabolic aspects. We will discuss them in detail later.

#### 2.2.3. Time-Resolved Proteomics

Time-resolved proteomics approach involves collecting samples at different time points during a biological process, infection, or treatment, and then analyzing the proteomic changes that occur over those time intervals. It is a powerful tool for deciphering temporal changes in disease progression [33], cellular responses [34], biological processes [35], and drug action [36]. These applications in *T. gondii* provide valuable knowledge about the dynamics of complex systems and molecular mechanisms. In addition, identifying proteins whose expression changes over time can contribute to the discovery of diagnostic or prognostic biomarkers. We will delve deeper into this discussion, with a specific focus on stage-specific proteomes, later on.

#### 2.2.4. Post-Translational Modifications (PTMs)

PTMs such as phosphorylation [37], glycosylation [38], and acetylation [39,40] play crucial roles in regulating various cellular processes in apicomplexan parasites, including *T. gondii*. These modifications affect protein activity, localization, and interactions, and contribute to the complexity of the proteome, thereby likely influencing developmental transitions, biology, and pathogenesis of these parasites. It provides a deeper understanding of the regulatory mechanisms that control the parasite’s ability to thrive in diverse environments and influence the processes within host cells. Later on, we will explore a detailed discussion of *T. gondii* PTMs and PTM crosstalk.

### 2.3. Recent Advancements in Proteomics Techniques

The advancements in MS have significantly improved its sensitivity, resolution, and throughput. High-resolution MS instruments like orbitrap and Q-TOF systems provide enhanced accuracy and dynamic range, enabling the identification and quantification of a wider range of proteins and PTMs. The recently developed Orbitrap/Astral dual analyzer mass spectrometer can handle five separate ion packets in parallel, leading to acquiring rates of up to 200 Hz [41]. This innovation substantially increases MS/MS scan speed, resulting in faster throughput, broader coverage, and higher sensitivity, along with accurate and precise quantitation in proteomics analysis. Here are some current hot topics in proteomics research, either currently in progress or poised for upcoming applications in *T. gondii* research.

#### 2.3.1. Single-Cell Proteomics

With the advance of MS, single-cell proteomics is experiencing rapid technological advancements and taking center stage [42]. These improvements include increased proteome coverage, which has grown from analyzing around 200 protein groups to over 1000 protein groups within individual mammalian cells [43]. Tandem Mass Tag (TMT) is typically ideal for studies requiring high multiplexing, compatibility with various sample types, and higher throughput. Junho et al. optimized the real-time search MS3-based TMT quantification method for the 126 single cells study; about 1000 out of the 1200 identified proteins were quantifiable [44]. In the 2023 ASMS meeting, Bruker highlighted the timsTOF Ultra’s capacity for single-cell proteomics, showing that the instrument can identify roughly 5000 proteins at the single-cell level and quantify more than 4800 proteins (https://www.nsmedicaldevices.com/news/bruker-introduces-timstof-ultra-mass-spectrometer-and-vistascan/# (accessed on 6 June 2023).

While single-cell proteomics publications in *T. gondii* applications are currently limited, we anticipate an upcoming surge in both applications and publications. This is expected to significantly enhance research on *T. gondii*. One of the main benefits of single-cell proteomics in *T. gondii* research is to strengthen our understanding of the heterogeneity of parasite–host interactions and provide insights into how different cells respond to the infection. It also reveals variations in parasite protein expression within the host environment. On the other hand, integrating single-cell proteomics with spatial information can help researchers understand the localized effects of *T. gondii* infection within infected tissues, which we will discuss further in the section on subcellular localization-associated proteome. Additionally, single-cell proteomics enables the tracking of temporal changes in protein turnover within individual cells. By introducing labeled amino acids, such as SILAC (Stable Isotope Labeling by Amino acids), into proteins over time, proteomics can potentially capture the intricate dynamics of protein turnover under different conditions, providing a detailed understanding of *T. gondii* host–pathogen interactions and cellular responses to infection.

#### 2.3.2. Data-Independent Acquisition (DIA) and High-Throughput Proteomics

DIA represents an emerging MS-based approach that effectively addresses the limitations associated with traditional data-dependent acquisition (DDA) methods. Through systematically fragmenting all discernible ions within a defined mass range, DIA offers several notable advantages, including expanded coverage of the proteome, enhanced precision in quantification, and the ability to detect proteins and PTMs at low abundance levels. Srinivasan et al. compared DIA with DDA methods for phosphoproteomics from nocodazole-treated and untreated U2OS cells. They found 15,548 unique site-localized phosphopeptides using DDA and 6817 using DIA. While DDA excels in identifying more unique analytes, DIA demonstrated better reproducibility, with approximately 66% of localized peptides consistently identified in at least 5 out of 10 replicates compared to 32% in DDA [45]. It anticipates that novel data analysis methods, such as machine learning, will leverage advanced MS instrumentation to enhance the capabilities of DIA-MS, enabling more comprehensive and accurate measurements of PTMs [46]. Furthermore, it supports high-throughput proteomics applications and allows for identifying, quantifying, and characterizing thousands of proteins within a single experiment. Combining offline high-pH reversed-phase peptide fractionation with short online LC gradients (180 samples per day) and DIA-MS enables near-complete coverage of the human proteome in just 4.5 h of LC-MS/MS analysis [47].

The use of DIA in *T. gondii* research is limited, with only a few applications. For instance, Alex et al. employed DIA to quantitatively analyze immunoprecipitated proteins from *T. gondii* cWT, cMut, and TIR1 parasites, leading to the measurement of over 2.5 thousand proteins [48]. DIA offers robust and reproducible quantifications across multiple samples, which are crucial for clinical implications. We anticipate that DIA will aid researchers in discovering novel proteins, protein isoforms, and PTMs in *T. gondii* studies. DIA is also well-suited for investigating the temporal dynamics of *T. gondii* proteome during different life cycle stages or in response to drug treatments. This is particularly valuable for identifying parasite proteins with rapid turnover rates that may play crucial roles in dynamic cellular processes or stress responses.

#### 2.3.3. Targeted Proteomics

Targeted proteomics, which includes techniques like selected reaction monitoring (SRM) and parallel reaction monitoring (PRM), is an active and rapidly advancing field in proteomics research. With advances in high-resolution mass spectrometers, targeted proteomics is gaining prominence in clinical and translational research [49]. It plays a crucial role in biomarker discovery and validation for various diseases. Researchers are developing targeted assays for specific proteins and PTMs relevant to disease. Nguyen et al. demonstrated the successful development of a workflow for the quantification of low abundance proteins involved in the UPR pathway, ranging from 4 to 103 amol of the LOQs for the targeted proteins were determined [50]. DIA approaches are also applied for the simultaneous quantification of multiple peptides within a predefined mass range in complex samples. Multiplexed targeted proteomics assays, such as TMT, are integrated with targeted proteomics for quantification [51]. Targeted proteomics is also applied in food safety and environmental monitoring, where it is used to quantify specific proteins and peptides relevant to these fields [52].

Targeted proteomics, recognized for its sensitivity, specificity, and reproducibility in quantitatively assessing and validating parasite protein expression levels, has been extensively employed in biomedical research and the pharmaceutical industry. Targeted proteomics often requires well-defined and characterized samples. Obtaining such samples from *T. gondii*, especially across different life stages (tachyzoite, bradyzoite, and oocyst), or during specific host interactions, each expressing a unique set of proteins, presents significant challenges. Despite its widespread use, the applications of targeted proteomics in *T. gondii* have been notably restricted, and there are scarce publications employing these techniques in the context of *T. gondii*. Nevertheless, this approach proves highly valuable for validating specific proteins/peptides, especially when antibodies are unavailable for alternative validation methods such as Western blotting. With continuous technological advancements and an expanding understanding of *T. gondii* biology, there is an anticipated increase in the feasibility of targeted proteomics. This advancement may facilitate a more extensive use of targeted proteomics to unravel specific aspects of the parasite’s proteome, including *T. gondii*‘s pathogenicity, host interactions, and drug targeting.

#### 2.3.4. Plasma Proteome

While fractionation and depletion techniques have made notable progress in improving coverage of the plasma proteome, the considerable 10 orders of magnitude dynamic range present in analyzed plasma samples remains a substantial challenge when applying proteomics to biomarker discovery and clinical applications in *T. gondii* infection. Even if researchers remove 95% of high-abundance proteins, including albumin and IgG, from plasma samples through high-abundance protein depletion, the remaining high-abundance proteins still significantly interfere with the detection of low-abundance proteins, such as secreted proteins and their PTMs. These low-abundance proteins might be crucial for host–parasite interactions but could undergo rapid degradation. Additionally, quantitative proteomics may suffer from accuracy and reproducibility issues, particularly in the low-abundance quantification of proteins. However, recent developments in MS technique shed light on these challenges; for example, using an innovative sample-enrichment kit called Mag-Net, a magnetic bead-centered procedure developed by Wu and colleagues [53], this study quantified more than 5000 plasma proteins from an extracellular vesicle-enriched sample in a single one-hour LC-DIA-MS/MS run, representing a significant advancement in the field [54].

#### 2.3.5. Top-Down Proteomics

Top-down proteomics is a powerful approach for studying intact proteins, including the characterization of protein isoforms and PTMs. With advancements in high-resolution mass spectrometers, particularly those equipped with Orbitrap or FT-ICR and data analysis tools, such as ProSight and TopPIC, top-down proteomics is well-suited for the characterization of complex PTMs, including glycosylation, phosphorylation, and acetylation, which are associated with key processes like invasion, gene expression, translation modulation, and immune evasion in *T. gondii,* at the protein level. Top-down proteomics also plays a crucial role in structural proteomics, which enhances our understanding of protein folding, conformational changes, and the study of PPIs at the intact protein level. There is growing interest in applying top-down proteomics to clinical research and diagnostics since it has the potential to identify *T. gondii* invasion and host interactions with specific protein isoforms, truncated forms, and PTMs for biomarker discovery. Recently, Melby JA et al. highlighted the effectiveness of the top-down proteomics method in characterizing protein isoforms (>200 kDa) diversity within single muscle cells and suggested its broader utility in understanding cellular heterogeneity and advancing precision medicine efforts [55]. It is also possible to integrate the results of hydrogen-deuterium exchange MS (HDX-MS) with top-down proteomics (Top-Down HDX MS) to gain a more comprehensive understanding of a protein’s structure, function, and dynamics [56]. For instance, HDX-MS can help us understand better how a protein’s conformational changes upon ligand binding, and these changes can be further analyzed using top-down proteomics to identify and characterize the modified protein forms (https://www.creative-proteomics.com/pronalyse/hdx-ms-and-how-it-works.html (accessed on 11 December 2023)).

Top-down proteomics, an evolving MS technology, requires specialized expertise and resources in terms of time and cost. Analyzing the complex and diverse proteome of *T. gondii* demands advanced sample preparation techniques and high-resolution mass spectrometers, posing challenges in confidently identifying intact proteins. As technology becomes more accessible and computational tools improve, the use of top-down proteomics in *T. gondii* research will likely increase. These techniques will certainly assist researchers in identifying potential therapeutic targets and specific antibodies against toxoplasmosis.

#### 2.3.6. Multi-Omics Integration

Multi-omics integration has also been applied to investigate host–pathogen interactions and infectious diseases. Jean Beltran et al. discussed proteomic methods and their application in studying host–pathogen interactions, highlighting how multi-omics approaches, including proteomics, genomics, transcriptomics, and metabolomics, contribute to a systems-level understanding of infectious diseases [57]. It has paved the way for the development of potential therapies and vaccines against toxoplasmosis and continues to be an active area of research. Leo et al. employed proteomics, transcriptomics, and pharmacoproteomics to analyze childhood acute lymphoblastic leukemia cell lines and their responses to oncology drugs, identifying correlations between molecular phenotypes and drug responses [58].

In *T. gondii* research, multi-omics integration has played a pivotal role. For instance, Kloehn et al. utilized multi-omics analysis to explore the distinct functions of subcellular acetyl-CoA pools in *T. gondii*. This approach unveiled a deeper understanding of the parasite’s physiological and metabolic adjustments [59]. Similarly, the study by Nie et al. employed global proteomic profiling and transcriptomics to understand the proteome-transcriptome correlation during *T. gondii* infection in cats. Their work shed light on the complex proteotranscriptomic reprogramming that mediates the dynamic interplays between *T. gondii* and feline tissues [60].

Machine learning approaches have been employed for multi-omics data analysis to uncover a deeper understanding of biological systems. Reel et al. reviewed different integrative machine learning methods used to analyze multi-omics data, which can aid in disease prediction, patient stratification, and precision medicine [61]. Analyzing these diverse data sources simultaneously can enable researchers to identify complex interactions and relationships that may not be apparent when analyzing each data type separately.

#### 2.3.7. Bioinformatics

Despite the breadth of ToxoDB, there exists a diverse set of external bioinformatics tools that could be integrated to construct a custom pipeline for reanalyzing existing data in *T. gondii* proteomics. Functional and comparative genomics have emerged as well-explored perspectives for analyzing proteomics, providing insights by linking functions to proteins in our dataset based on knowledge from model organisms [62]. While pipelines for analyzing proteomics datasets from various perspectives, including epigenetics, have been developed [63], the majority of studies focus on predicting new antigens for diagnosis or vaccine development [64,65,66,67,68]. Multi-epitopes antigens are designed using bioinformatics tools like the ANTIGENpro database (http://scratch.proteomics.ics.uci.edu (accessed on 11 December 2023)), NetCTL 1.2 server (http://www.cbs.dtu.dk/services/NetCTL/ (accessed on 11 December 2023)), IFN epitope server (http://crdd.osdd.net/raghava/ifnepitope/scan.php (accessed on 11 December 2023)) and the ABCpred server (http://www.imtech.res.in/raghava/abcpred/ (accessed on 11 December 2023)). Notably, Goodswen et al. have recently introduced an advanced bioinformatics pipeline that integrates proteomics data from ToxoDB along with various external tools to predict potential vaccine candidates [69].

One of the aims of parasite bioinformatics studies at the proteomics level is to determine the protein–protein interactome [70]. A significant proportion of proteomics studies in the field attempt to propose an interaction network from experimental results. Although it is possible to predict these interactions from an input dataset using the STRINGdb tool integrated into ToxoDB, this prediction typically fails in the absence of additional information for uncharacterized genes [71]. Recently, a gene regulatory network analysis successfully predicted interactions between genes, integrating with proteomics data and improving *T. gondii* gene annotation [72]. The future challenge for this approach is to integrate omics data from the host to determine host–pathogen interaction at the protein level [73]. Additionally, available bioinformatics tools for network analysis, such as WGCNA [74] and BioNetStat [75], could be implemented. Finally, further studies need to focus on *T. gondii* proteome functional annotation since approximately 40% of *T. gondii* gene products remain uncharacterized [76]. In this sense, exploring bioinformatics approaches available for other pathogens models, such as kinetoplastids, which have notably improved sensitivity in genome and proteome annotations (https://github.com/sradiouy/DARK, https://doi.org/10.1016/j.molbiopara.2015.09.002, https://doi.org/10.3389/fgene.2022.1020100 (accessed on 11 December 2023), could be beneficial.

## 3. Interactome Analysis

Interactome analysis aims to map and characterize PPIs between *T. gondii* proteins and host proteins, which can identify key protein complexes and pathways involved in host–parasite interactions [77,78,79]. Xia J et al. investigated the human interactome of the *T. gondii* rhoptry protein ROP18 (TgROP18) and identified the targets of ROP18I and ROP18II, highlighting their association with immune response and apoptosis [3]. Matthew et al. conducted a proteomic analysis of host cells infected with different types of parasites expressing MAF1b, MAF1a, and a non-functional mutant of MAF1b. Among the 13 proteins uniquely enriched in the MAF1b pull-down experiments, two host proteins—TOM70 and HSPA9—were identified as essential mediators of the Host Mitochondrial Association (HMA) phenomenon driven by MAF1b [80].

Below are several common sample preparation techniques and approaches employed for studying PPIs.

### 3.1. Affinity Purification

In this approach, *T. gondii* proteins are tagged with a specific affinity tag (such as FLAG or HA tags) and expressed in the parasite. The tagged proteins and their interacting partners can then be selectively isolated using affinity purification techniques, followed by identification using MS. Yakubu et al. utilized affinity purification combined with proteomics to reveal that Protein Arginine Methyltransferase 1 (PRMT1) significantly contributes to arginine monomethylation in *T. gondii* [28]. Anghel et al. applied differential affinity chromatography followed by MS to investigate cellular and molecular targets of nucleotide-tagged ruthenium complexes in *T. gondii* and *Trypanosoma brucei* [81]. Müller et al. employed differential affinity chromatography coupled with MS to identify common binding proteins for an antimicrobial peptide in *T. gondii* [82]. Li et al. conducted a global proteome analysis of lysine crotonylation (Kcr) in *T. gondii* using LC-MS/MS and an immune-affinity method. They identified thousands of Kcr sites on diverse proteins, suggesting a widespread involvement of Kcr in various biological processes [83]. Sun et al. demonstrated the significance of immunoproteomics and MALDI-TOF MS in identifying antigenic proteins of *T. gondii* RH strain that 18 immunoreactive proteins had been identified and recognized by human immunoglobulin G (IgG). Notably, these proteins did not show reactivity with negative-control sera from healthy individuals without *Toxoplasma* infection [84].

### 3.2. Proximity Labeling Techniques

By labeling molecules that are close to a specific protein or molecular complex of interest, the labeled proteins can be isolated and identified using MS. Proximity labeling techniques offer a high-throughput approach to studying various biomolecular interactions with spatial and temporal precision. Shkel et al. utilized enzymes to covalently label nearby biomolecules, enabling their identification using MS. Their advantage lies in capturing weak or transient interactions, making them crucial for investigating organelle interactomes and macromolecular complexes [85]. Back et al. used proximity labeling to identify novel IMC proteins enriched in daughter buds and revealed that IMC29 plays an important role in *Toxoplasma* endodyogeny [86]. Cygan et al. employed proximity labeling to identify proteins at the host-cytosolic side of the PVM. The study identified specific parasites (GRA61, GRA62, GRA63) and host proteins (PDCD6IP/ALIX, PDCD6, CC2D1A, MOSPD2) in PVM [87].

### 3.3. BirA*-Mediated Proximity-Dependent Biotin Identification (BioID)

BioID is a specific proximity labeling technique that uses a mutant form of the biotin ligase enzyme (BirA*) as the labeling enzyme. The protein of interest is fused to BirA*, and when the fusion protein is expressed in cells, BirA* biotinylates lysine residues on proteins within its spatial proximity. These biotinylated proteins can be isolated using streptavidin-based purification and then identified using MS [88]. Engelberg et al. used BioID technology and identified four architecturally distinct basal complexBC subcomplexes, and key proteins including BCC0, BCC4, and MORN1, highlighting their roles in different aspects of cell division [88]. Proteins associated with coiled-coil structures and involved in signaling functions have been detected in the apical annuli and are linked to inner membrane complex (IMC) sutures [89]. Nadipuram et al. applied BioID technology to uncover novel dense granule proteins secreted by *T. gondii* bradyzoites and identify previously unknown proteins involved in chronic infection and cyst formation [90]. Tu et al. employed BioID to identify clusters of proteins associated with dense granules, cyst matrix, and the cyst wall, enhancing the understanding of cyst wall composition [91]. Song et al. utilized BioID to identify more than 300 proximal interacting proteins of calmodulin (CaM) in *T. gondii*. CaM was found to play essential roles in tachyzoite proliferation, invasion, and egress, with potential functions related to ion binding and oxidation reduction [92].

### 3.4. Ascorbate Peroxidase-Mediated Proximity Labeling (APEX)

One notable benefit of APEX compared to traditional BioID is its considerably faster labeling rate, operating within minutes as opposed to hours. When combined with quantitative proteomic methods, this helps detect the rapid alterations in protein interactions over time or as a reaction to cellular disturbances [93,94]. With BioID and APEX techniques, Pan M et al. identified 46 proteins, including 20 known and 26 new GRAs. These GRAs, mainly in coccidian parasites, might not be vital for in vitro growth but could play roles in animal infections [95].

### 3.5. Yeast Two Hybrid (Y2H)

Yeast two-hybrid (Y2H) assays are useful to identify PPIs in cells. Two proteins, one “bait” and one “prey,” are put into yeast cells. If there is an interaction, it is proven by the activation of reporter genes. Using Y2H assays, Lai et al. discovered significant interactions between *T. gondii* surface antigens (SAG2 and SAG1) and specific human proteins. They identified 20 and 39 positive clones interacting with SAG2 and SAG1, respectively. Notably, *Homo sapiens* zinc finger protein strongly interacted with SAG2, while *Homo sapiens* lysine-rich coil-coiled protein showed a strong interaction with SAG1 [96,97].

Interactome analysis using the abovementioned techniques has significantly contributed to our understanding of host–parasite interaction networks, virulence factors, and intracellular signaling pathways, and facilitated the identification of potential therapeutic targets for combating toxoplasmosis.

## 4. ToxoDB in *T. gondii* Proteome

**Figure 3:**
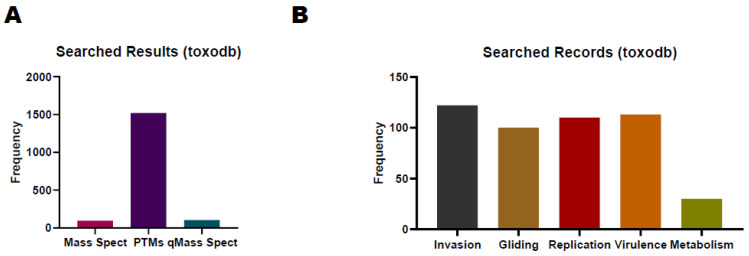
Summary of proteomics and biological processes searching results in ToxoDB. (**A**) *T.gondii* proteomics research counts in ToxoDB. (**B**) Search results of typical biological processes of *T.gondii* in ToxoDB.

The ToxoDB database (https://toxodb.org/toxo/app/ (accessed on 10 October 2023)) is a valuable resource for the proteome study of *T. gondii*, allowing researchers to access experimental data related to *T. gondii* proteins, which include MS data, protein expression profiles, and post-translational modification information [98]. According to the current release, the ToxoDB-65_TgondiiME49_AnnotatedProteins database, which contains 8322 protein-coding genes, can be used for proteomics database searching. Researchers can also cross-reference proteomic data with other omics data to gain a more comprehensive understanding of *T. gondii* biology. There are 96, 1521, and 104 records about *T. gondii* research for MS, PTMs, and quantitative MS, respectively, in ToxoDB (Figure 3A). We also list the search results by inputting the keywords of different biological processes such as invasion, virulence, replication, metabolism, and gliding (Figure 3B). Comparing identified proteins and expression levels with those available in ToxoDB, researchers can understand when and where the proteins of interest are expressed in different stages of the parasite’s life cycle. The visualization tools and bioinformatics resources on ToxoDB can help identify integrated data patterns, correlations, and potential functional perceptions. For example, ROP18, an essential virulence factor, plays a pivotal role in the parasite’s capacity to initiate infection and induce illness in the host. This protein possesses serine/threonine kinase activity, enabling it to phosphorylate host cell proteins. Consequently, ROP18 can manipulate host cell functions and immune responses. ToxoDB contains 36 records related to ROP18 in the context of MS, 5 records concerning PTMs, and 4 records for Quantitative MS in studies involving *T. gondii*. Researchers can utilize these records to construct protein/peptide libraries or seamlessly integrate them into other omics projects. With more data imputed into the database, ToxoDB will play many more roles in the integration of proteomics with genomics data, providing various bioinformatics tools and resources to assist researchers in data analysis.

### 4.1. Stage-Specific Proteomes

The first *T. gondii* proteome was generated from the tachyzoite stage [99]. In that work, three proteomic platforms were designed: two-dimensional electrophoresis, one-dimensional electrophoresis gel coupled with LC-MS/MS, and MudPIT analysis. By combining these methods, the authors identified 2252 non-redundant expressed proteins, which were approximately 29% of the annotated genes. Notably, 57% of the genes exhibiting evidence of transcription through the Expressed Sequence Tag method failed to yield detectable peptides, suggesting potential factors such as low translation levels, protein instability, transient protein expression, or even the existence of non-coding RNAs. In contrast, a comparison of the proteins identified in this study with mRNA expression levels, determined by microarray analysis, revealed a substantial number of proteins were detected even in cases when mRNA expression levels were negligible or minimal. This observation suggests the presence of highly stable proteins over time, with 204 and 632 instances in cases of minimal mRNA expression, respectively. Additionally, peptide identification aided in defining or confirming the exon–intron predictions of the annotated genes. The incorporation of proteome data from several laboratories over time, each with different objectives and strategies, contributed to raising the number of proteins identified in the tachyzoite stage to 5325 [100]. Another proteomic study in EuPathDB identified approximately 35% of the total protein-coding genes and about 50% of the total proteins for ME49 [101]. Interestingly, this analysis added 289 loci via RNA-seq and proteomics that were not previously identified.

In 2013, the proteome of partially sporulated oocysts was published [100]. The generation of the oocyst-stage proteins was of high relevance due to the limited number of studies carried out on this stage. Moreover, the lack of identification of specific stage proteins hindered progress on this important stage for environmental dissemination of *T. gondii*. The analysis compared the oocyst proteome with transcriptomic data obtained in unsporulated (day 0) and sporulated (day 10) oocysts. At day 0, the analysis identified proteins associated with upregulated genes related to metabolism, cellular transport, and cell fate, while day 10 showed enrichment in proteins associated with protein synthesis, cell rescue, defense, and virulence. On the other hand, the comparison of this proteome with that of tachyzoites identified 154 oocyst-specific proteins, many associated with hypothetical genes. Oocyst-specific functional genes tend to be enriched in metabolic functions, comprising the largest category. This is followed by proteins involved in processes related to cell rescue, defense, virulence, energy production, and protein fate. In comparison to tachyzoites, oocysts have a greater capability of de novo amino acid biosynthesis and are well equipped to fuel the glycolysis/gluconeogenesis and tricarboxylic cycle (TCA), including the expression of Eno1 (enolase 1 gene) in the unsporulated oocyst, which is also expressed in bradyzoite.

The generation of a bradyzoite stage proteome posed more challenges. In the in vitro differentiation process, only less than 80% of *T. gondii* tachyzoites differentiate into bradyzoites. For this reason, Garfoot et al. [102] conducted experimental infections in CBA/J mice using the Me49 strain. In the in vivo model, in contrast, 100% of *T. gondii* differentiated into bradyzoites were obtained; however, their extraction was carried out from brains (brain bradyzoite) of infected mice at 21–90 days post infection (dpi), requiring a process of purification in dextran to obtain larger amounts of peptide reads. In total, 1683 *T. gondii* proteins were mapped in the bradyzoite brain proteome, and 893 proteins when duplicates were taken into account. Only 366 proteins were identified as common to all time points analyzed. Part of the sample was used to generate a transcriptome. Most of the genes that encode the 366 proteins exhibited high levels of expression. However, 100 of the 366 proteins presented a low level of gene expression during chronic infection. This could be due to short-lived transcripts but long-lived proteins. An interesting finding from the RNA-seq analysis is that the transcriptomic profile remains constant in the period from 28 to 120 days of the analysis.

### 4.2. PTM Proteomes

The proteomes of tachyzoites, bradyzoites, and oocysts can offer information about the genes of interest that potentially provide prior knowledge about their expression levels in the different stages. However, proteomes that investigate various PTMs can provide valuable insights for researchers interested in the study of a specific gene. There are more than 400 protein PTMs, which in turn present crosstalk with each other. In *T. gondii*, information from a PTM could help formulate a hypothesis regarding the function and/or regulation of the protein of interest. Knowing which residues have incorporated specific PTMs provides essential information for generating site-specific mutants. In ToxoDB, there is data obtained from three PTM proteomes: phosphorylation, acetylation, and ubiquitinylation. Beyond the scope of ToxoDB, other more specific proteomes analyze PTMs in various contexts, including histone PTMs [103], acetylation in *T. gondii* deficient in acetyl-coA [59], crotonylation [83], phosphorylation in kinase mutants or different stages [37,104,105,106,107,108,109], lipidation [110,111], Cysteine S-nitrosylation [112]. In this section, we will only focus on the PTMs obtained in the proteomes available in the ToxoDB.

#### 4.2.1. Phosphoproteome

In 2011, Treeck et al. [113] generated the first phosphoproteome of *T. gondii* and *Plasmodium falciparum*. The objective was to obtain information about which proteins could regulate their activity through this PTM. Although it is common to both parasites, *T. gondii* possesses several kinases, including protein kinase A, protein kinase G, rhoptries, calcium-dependent kinases, etc., which play important roles in various biological processes, such as invasion, egress, DNA and parasite replication, pathogenesis, differentiation, and energy metabolism [114,115,116,117,118,119]. The *T. gondii* phosphoproteome was generated based on two sources: intracellular tachyzoites and tachyzoites purified from cell cultures in which secreted parasite proteins were discarded [113]. In total, 2793 (intracellular) and 3506 (purified) phosphorylated proteins were identified, detecting 12,793 and 24,298 phosphorylated sites, respectively. By the comparison of the intracellular and purified parasites, about 50 proteins were detected that are phosphorylated within the host cell, in turn, suggesting that most of the exportable proteins (with signal peptide) are phosphorylated in the tachyzoite.

#### 4.2.2. Acetylome

Acetylation is a PTM that was initially studied in histones and its role in chromatin modulation. However, it has been observed that acetylation regulates the activity and function of many non-histone cytoplasmic and nuclear proteins [120]. The acetylation primarily occurs on the lysine residues, and this PTM can be reversible (Nε-acetylation and O-acetylation) or irreversible (Nα-acetylation). Other residues including serine, threonine, and histidine also can undergo acetylation [121,122]. In *T. gondii*, it has been shown that the reversible acetylation process, mediated by lysine acetyltransferases (KAT) and lysine deacetyl transferases (KDAC), can be excellent therapeutic targets [123]. Among the KDAC inhibitors, also called histone deacetyl transferases, identified are apicidin, FR235222, hydroxamate-based compounds, panobinostat, JF363, tubastatin, SAHA, and MC1742 [124,125,126,127,128,129,130]. Additionally, a KDAC activator, resveratrol, has been described [131].

The *T. gondii* acetylome identified 411 lysine acetylation sites across 274 tachyzoite proteins [132], data present in the ToxoDB. The vast majority of acetylated proteins are associated with translation, energy metabolism, chromatin-associated proteins, and stress response. The study detected 25 proteins associated with cellular metabolism in addition to five histones. In the acetylation process, the donor of the acetyl group is acetyl-CoA. In a subsequent study, the acetylome of a *T. gondii* double mutant was analyzed. The double mutant where the ATP citrate lyase gene was deleted (ΔACL), together with an inducible knockdown (KD) of the acetyl-CoA synthetase [59], is inviable when KD is induced. The acetylome of these tachyzoites shows hypoacetylation of proteins involved in metabolism and histones, among others.

An acetylome of three *T. gondii* strains was also generated for comparative analysis [133]: the virulent strains RH, PYS (Chinese 1), and the avirulent PRU. Based on the analysis of the two replicates for each case, 457 acetylated proteins for RH and 188 acetylated proteins for PYS were identified. In the case of the RH strain, the top 6 of the KEGG pathways were metabolic pathways, biosynthesis of secondary metabolites, biosynthesis of antibiotics, microbial metabolism in diverse environments, ribosome and carbon metabolism. In the PYS strain, the top six of the enriched KEGG pathways were metabolic pathways, biosynthesis of secondary metabolites, biosynthesis of antibiotics, carbon metabolism, microbial metabolism in diverse environments, and the ribosome. It is noted that both acetylomes are very similar. The PRU acetylome yielded 134 acetylated proteins, exhibiting a KEGG pathway profile very similar to that of RH and PYS. However, acetylated proteins from RH and PYS were more enriched in the pyruvate metabolism pathway compared to acetylated proteins from the PRU strain. Between RH and PYS, 26 differentially acetylated proteins (DAP) were observed: 2 upregulated and 24 downregulated in PYS, including histone-acetyl-transferase and glycyl-tRNA synthase. Both enzymes play roles in stress tolerance and proliferation, which are key features in parasite virulence.

#### 4.2.3. Ubiquitin Proteome

Ubiquitinylation is a process where one or several molecules are conjugated to a protein. Ubiquitin is composed of 76 amino acids and has a molecular mass of approximately 8.6 kDa. Its structure is highly conserved in the eukaryotic lineage. If the binding to the target protein occurs primarily through lysine 48 of ubiquitin, with lysines 6 and 33 playing a minor role, this protein will undergo degradation. On the other hand, binding by lysine 63 of the ubiquitin leads the target protein to different biological roles such as DNA repair, chromatin silencing, signaling activation, protein trafficking, and receptor endocytosis [134]. The ubiquitinome of intracellular tachyzoites of *T. gondii* showed 454 ubiquitinylated proteins and 800 sites [135]. Ubiquitinylated proteins are involved in different subcellular locations and functions, but this PTM was mainly detected in proteins of the cytoskeleton, inner membrane complex (IMC), and the nucleus, including histones. One interesting aspect of the study was the regulation of ubiquitinylation in *T. gondii* proteins throughout the cell cycle. The ubiquitinome of the extracellular tachyzoite comprised 346 proteins, of which 51 were typical of the extracellular stage, including glycolytic enzymes, matching the metabolic differences between the extracellular and intracellular stages [136].

Ubiquitination, associated with the degradation of cell cycle proteins, is a thoroughly documented mechanism in eukaryotic cells, where proteins undergo turnover to fulfill specific roles during the progression of the cell cycle. However, this aspect may differ significantly in *T. gondii*. While some ubiquitinated proteins from the replisome were detected in the *T. gondii*, the notable absence of proteins that should undergo ubiquitination throughout the cell cycle, such as cyclins and cyclin-dependent protein kinases, suggests that the regulation of the cell cycle in the parasite may have unique characteristics [135].

### 4.3. PTM Crosstalk and Proteome

A study on combined methylation, phosphorylation, and acetylation of proteins conducted in a lung cancer model showed a large number of proteins that presented the three PTMs [137,138]. Taking a functional perspective, PTMs increase the diversity of functional units of protein origin within a cell. PTMs are still a field with great potential for future exploration in *T. gondii.* A crosstalk study with ubiquitination and the other PTMs showed that less than 10% of the proteins overlapped between ubiquitin and arginine methylation, 21% exhibited a combination of acetylation with ubiquitin, 25% of the SUMO proteome was ubiquitinated, while 78% of the phosphorylated proteins were also ubiquitinated [135]. Among the proteins detected with combinations of different PTMs, the histone acetyltransferase EP300 and the chaperone Hsp90 [137] stand out.

#### 4.3.1. Histones

Over several decades of research, it has become evident that various PTMs on histone proteins engage in intricate crosstalk, ultimately influencing the regulation of gene expression. This intricate interplay of histone modifications has been characterized as a “histone code,” which specific proteins can interpret, providing in conjunction with the substitution of different histone variants, a means for epigenetic regulation [139]. Understanding the roles of PTMs is also complicated by the fact that the same writers and readers of marks also write and read PTMs on other proteins [140].

Protozoan parasites, such as *T. gondii*, are no exception to this trend. The use of high-throughput mass spectrometry is enabling the systematic dissection of histone modifications and core markers involved in epigenetic regulation [141]. Numerous residues have been identified for modification, particularly within the histone tails. However, it is worth noting that various residues within the histone globular domains can also undergo diverse modifications, encompassing processes such as acetylation, phosphorylation, ubiquitination, methylation, and, lately, crotonylation and 2-hydroxyisobutyrylation [141]. These last newly described marks have demonstrated their crucial significance in regulating global transcription in mammalian cells. Consequently, in *T. gondii*, they may exert a noteworthy influence on the transcription process [141].

**Figure 4:**
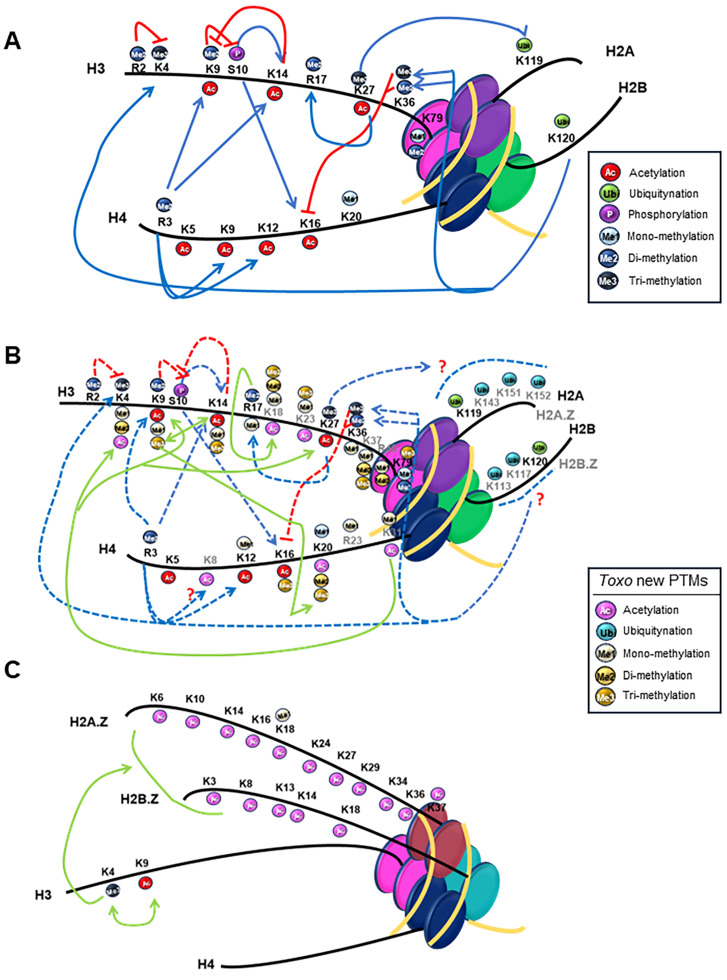
Histone PTMs crosstalk. (**A**) Mammals and other eukaryotes: schematic representation of a nucleosome particle with the N-terminal tails of H3 and H4, and C-terminal tails of H2A and H2B histones. PTMs are represented in colored symbols as specified in the box. Crosstalks that have been studied are shown in blue arrows when one PTM activates another and in red lines when it is repressive. (**B**) Schematic representation of a *Toxoplasma gondii* nucleosome particle, where canonical H3, H4, H2A, and H2B are shown as in (**A**). H2A.Z and H2B.Z *T. gondii* C-terminal tails are represented in grey letters with the lysine residues, which were shown to be ubiquitinated, although H2A and H2B canonical ubiquitination are also conserved. Amino acidic residues are shown in black letters when conserved in *T. gondii*, while novel residues are shown in grey letters. The PTMs that are conserved in *T. gondii* follow the same color key as in (**A**), while the differential *Toxoplasma* PTMs are shown in the box entitled “*Toxo* new PTMs” (the same for (**C**)). Although not much work has been conducted on *T. gondii* crosstalks, many of them can be inferred because of the conservation of the residues, and are marked in dotted lines. When a different residue may be involved in the crosstalk, but has not been proved, it is represented with dotted lines plus a question mark. New *T. gondii* crosstalks that have been observed are shown in green lines. (**C**) Schematic representation of a *Toxoplasma gondii* nucleosome particle in which canonical H2A/H2B histones have been replaced by the H2A.Z/H2B.Z DVN, where the N-terminal tails with the PTMs are represented. Only the PTMs in H3 that have been observed to crosstalk with this DVN are shown.

There is a high frequency of lysine residues in histones, and these residues are often modified by PTMs [103]. Among the histones, H3 and H4 PTMs are widely distributed not only in unicellular organisms, like protozoan parasites, but also in multicellular eukaryotes, and many of the amino acid residues that carry certain PTMs are highly conserved [140]. Figure 4A shows the different histone marks and their crosstalk in the mammal model as described. Furthermore, histone marks identified in *T. gondii* PTM proteomic analysis were highlighted to compare (Figure 4B). In the figure, possible crosstalk conservation, similar to those observed in mammals, is indicated with punctuated lines, although some new marks are also detected and the crosstalks are marked as questions. As observed, almost all the marks are present, and although not much research has been conducted in this area in the parasite, their crosstalk can be inferred. As shown in the figure, in *T. gondii*, the N-terminal tails of H4 and H3 are highly acetylated and methylated regions [86]. Interestingly, some of the residues that are acetylated can also be methylated, like lysine 12 (H4K12me) or lysine 16 when not acetylated, was found trimethylated (H4K16me3) [103]. Another example is H3K9, which was also found methylated and acetylated. While mono methylation and acetylation are active marks, di or tri methylation is often repressive and found in subtelomeres and centromeres [142]. Acetylation on H4K31 has also been reported in *T. gondii* and *P. falciparum* and this residue can also be mono-methylated in a mutually exclusive manner [132,143]. In *T. gondii*, this residue was found to be acetylated at the promoter of nearby active genes associated with H3K4me3 and H3K9ac marks (Figure 4B). At the same time, it was mono-methylated in the core body of the gene, and it inversely correlated with gene expression, suggesting it would be a repressive mark as opposed to H4K31ac [143]. Some PTMs not found in other species for this histone were found, like H4R23me, in the globular domain and methylation, both di and monomethylation on the C-terminal tail, on R78 [103].

Another important modification on lysine residues is ubiquitination, but few PTMs occur on the same lysine residues as ubiquitin. One exception is H3K24, which is modified by ubiquitin, acetylation, and mono- and trimethylation, implicating it as an important regulatory site [135]. Apart from this residue, only H3K116 was found to be ubiquitinylated, although other modifications occur at substoichiometric levels only detectable using an enrichment strategy [135].

There are also few PTMs in H2A.X, but two were found in the C-terminal tail, acetylation of K128 that has not been described, and the phosphorylated S132 [103], which corresponds to S139p in humans with a conserved function for TgH2A.X in response to DNA damage [144]. In contrast, H2A.Z displays several PTMs, with many acetylations in the N-terminal tail standing out. Within the first 40 amino acids, there are 10 lysines (K6, K10, K14, K18, K24, K27, K29, K34, K36, and K37), all of which are acetylated. Additionally, lysine K18 could also be methylated, leading to various PTM combinations with acetylated residues [103].

A similar situation arises for H2B histones, as canonical H2B possesses only one acetylation on the N-terminus at K4, whereas H2B.Z exhibits multiple modifications in the N-terminal tail, including acetylation on lysines 3, 8, 13, 14, and 18 [103]. Another study using an H2BK120 ubiquitin-specific antibody detected reactivity with *T. gondii* H2B and H2B.Z supporting the presence of ubiquitin–histone conjugates in *T. gondii* chromatin that could have conserved functions [135]. Histone H2B.Z is unique to apicomplexan parasites and has been described to conform to a double variant nucleosome (DVN), hyperacetylated in the N-terminal tail and associated with transcriptional activation (Figure 4C) [39,142,144,145,146,147]. Recent research has examined acetylations in the H2B.Z N-terminal tail lysines and their significance in various biological processes in *T. gondii* [39]. This study revealed that the inability to regulate this N-tail positive charge patch when no acetylation is possible, produced a reduced in vitro replication, a heightened differentiation rate, an increased sensitivity to DNA damage, and, in an in vivo model, a complete loss of *T. gondii* virulence [39].

As mentioned above, not much research has been conducted to date on histone crosstalks in *T. gondii*. Nevertheless, some conclusions have been made, taking into account results when one mark can be sufficient to elicit a specific biological output while in other cases, multiple marks are required [148] (Figure 4B). For example, it has been proposed that gene activation needs the dual signature of H3R17me2 and H3K18 [149]. In this work, the *T. gondii* arginine methyltransferase TgCARM1 was shown to work in concert with the acetylase TgGCN5-A, whose substrate preference is H3K18. Another example is the redundancy or multiplicity of sumoylation sites in the parasite histones, as suggested by Bougdour et al. [148].

Also, the transcription-associated PTMs H3K4ac, H3K9ac, and H3K27ac were found to be associated with H4K31ac in euchromatic regions and opposed to the heterochromatic regions revealed by the repressive marks H3K9me3 and H4K20me3 [143] (Figure 4B). In another study, the DVN conformed by H2A.Z and H2B.Z, which are both hyperacetylated in the N-terminal tails, was found in association with the activatory mark H3K4me3 [146]. In turn, H3K4me3 was found along with H3K9ac in most of the *T. gondii* genes that are expressed (Figure 4C) [148].

Another well-characterized example first in yeast but then in other eukaryotes is the requirement of histone H2B monoubiquitination for proper H3K4 and H3K79 methylation [139,150,151]. Although ubiquitination was detected on TgH2AK119Ub and TgH2BK120Ub [135], to date it has only been speculated that acetylation and ubiquitination may regulate the differential localization of H2A.Z and H2B.Z on active and silent genes in *T. gondii* [142], and so it is represented as a question in Figure 4B.

#### 4.3.2. Hsp90 PTM Crosstalk

The Hsp90 chaperone was shown to be simultaneously acetylated and phosphorylated in the lung cancer model [137]. This protein is central since its main functions are to assist important proteins in biological processes such as DNA replication, regulation of gene expression, proliferation, etc., in both stressed and non-stressed cells. The Hsp90 protein network includes hundreds of proteins, mainly transcription factors, and kinases, but also proteins associated with chromatin, metabolism, translation, and DNA damage [152,153,154]. Hsp90 can form different complexes, one of them called the Hsp70/Hsp90 cycle. It may include other co-chaperones, the hsp40 type I being the one that initiates the recruitment of the client protein. The Hsp70/Hsp90 cycle is conserved in *T. gondii* [154]. Recently, the proteome of Hsp90 and Hsp40 type I in *T. gondii* was analyzed and referred to as Tgj1 [79]. This study allowed defining a putative PPI network for both chaperones as part of the Hsp70/Hsp90 cycle and in their independent functions between them. Within the Hsp70/Hsp90 cycle, the enriched pathways were translation, cell redox homeostasis, and protein folding. The PPI associated with Hsp90 not related to the Tgj1-Hsp90 axis showed mainly interactors related to protein folding, RNA processing, cell signaling, and transcription. This shows the important role of this chaperone in a wide diversity of biological processes of *T. gondii*. The proteomes related to PTMs show that *T. gondii* Hsp90 presents phosphorylations, acetylations, ubiquitinylation, and even a monomethyl arginine modification (ToxoDB_TGME49_288380). Backe et al. describe the role of the different PTMs in Hsp90 in the review [155]. Based on the role of the PTMs present in Hsp90 of other organisms, a proposed role for the *T. gondii* Hsp90 PTMs counterpart can be deduced [78]. Briefly, phosphorylation of Hsp90 in yeast and other species may affect its binding to certain client proteins or even its affinity for ATP. In other cases (e.g., Hsp90 T101), it may promote kinase client activation. Hsp90 phosphorylation in turn is associated with the cell cycle and in some cases promotes cell proliferation [156,157]. Therefore, these PTMs could similarly affect *T. gondii* Hsp90. Phosphorylation of threonine 5 and 7 on Hsp90 are associated with double-strand break repair [158], but these modifications were not detected in *T. gondii*. The phosphorylation of residues S231 (T220 in *T. gondii*) and S263 is related to telomerase activity [159]. Similarly, acetylation of Hsp90 leads to a loss of affinity for ATP and a decrease in its binding to the client protein and co-chaperones [155]. *T. gondii* presents two acetylations in the acetylome (K384 and K559). Acetylation of lysine K384 in human Hsp90 affects the interaction with its client protein, the receptor tyrosine kinase ErbB2 [160]. Acetylation at K558 (TgK559) could be associated with the export of Hsp90 to the extracellular space [161]. The ubiquitinylation of Hsp90 seems to be associated only with its degradation. CHIP is one of the co-chaperones associated with the Hsp70/Hsp90 cycle, which negatively regulates the pathway. This CHIP protein is an E3 ubiquitin ligase and binds to Hsp90. However, phosphorylated forms of Hsp90 decrease their association with CHIP, suggesting a crosstalk between both PTMs [162].

## 5. Subcellular Localization Associated Proteome

For researchers delving into an uncharted protein of interest, one of the key considerations for its characterization and the potential revelation of its function lies in determining its subcellular localization. This becomes even more crucial in omics studies, where a multitude of genes or proteins are involved, and establishing associations among them often requires them to inhabit the same cellular compartments. Beyond proteomes focused on subcellular locations, some proteomics studies also analyze the presence of *T. gondii* proteins within specific cellular structures. For example, *T. gondii* conoid proteome was performed [163]. This structure is relevant for invasion in *T. gondii* and closely related parasites, but is absent in other apicomplexans, such as *Plasmodium* spp. In this work, 200 proteins located in the cytoskeleton conoid and the apical region of *T. gondii* were identified. Another work that provides very interesting data is the proteome of extracellular proteins and vesicles (exosomes/ectosomes) [164]. In this work, 512 proteins were detected in the excretory/secretory, 210 in the ectosome, and 285 in the exosome fraction. The authors also analyzed the supernatant obtained from the purification of vesicles, identifying 421 proteins in that fraction. Another study, in which data has been uploaded to ToxoDB, detected 346 proteins in extracellular vesicles [165]. As expected, proteins from the secretory organelles micronemes, rhoptries, and dense granules were detected. However, kinases, heat shock proteins, and even nuclear proteins, including histones, were also identified, showing the wide diversity of proteins released by some pathways, which could have an implication not yet studied in toxoplasmic infection. Another particular proteomic study is the analysis of the proteins present in the wall of the *T. gondii* cyst [166]. In this study, numerous anticipated proteins such as CST1, BPK1, MCP4, MAG1, GRA2, GRA3, and GRA5 were identified, and new components were also discovered. In one case (CST2), its null deletion altered the virulence and lost the cyst formation. Based on the importance of rhoptry in the invasion process a proteomic analysis of rhoptries was performed [167]. The authors identified 38 novel rhoptry proteins.

*Toxoplasma* has a single mitochondria that is very relevant during the tachyzoite stage. Researchers observed that bradyzoites lack a functional TCA and respiratory chain [168]. The mitochondrial proteome was generated based on two proximal labeling systems already described above: one by mBirA and another modified for mitochondria using the plant ascorbate peroxidase (APEX) system [169]. The proteome data were uploaded to ToxoDB. In total, researchers identified 421 proteins: 213 in the APEX samples and 369 in the mBirA system. Notably, 36% were hypothetical and 33% were never defined in a role or location associated with mitochondria. Although some of them presented another non-mitochondrial location by other techniques, in the majority their location was confirmed. Among the new proteins identified, the authors focused on one called TgApiCox25, which was shown to be a component of the cytochrome c oxidase complex.

In the ToxoDB, two *T. gondii* proteomes analyze the components through subcellular fractionation. One was generated by the Laboratory of Dr. Silvia Moreno (Center for Tropical and Emerging Global Diseases, Department of Cellular Biology, University of Georgia, Athens, GA, USA). Another is based on a fractionation called hyperlexed localization of organelle proteins by isotope tagging (hyperLOPIT). LOPIT is based on protein correlation profiling, in which all proteins from a subcellular location can be marked by tandem mass tags followed by mass spectrometry [170]. In the case of *T. gondii* HyperLOPIT, an iodixanol density gradient was used to separate the subcellular compartments [32]. Tagged peptides from the different fractions of the gradient were analyzed. A subcellular localization map was generated based on the following compartments: apical 1, apical 2, micronemes, rhoptries 1, rhoptries 2, dense granules, IMC, tubulin cytoskeleton, Plasma Membrane (PM)—peripheral 1, endomembrane/vesicles, PM—peripheral 2, PM—integral, Golgi, endoplasmic reticulum (ER) 1, ER 2, apicoplast, mitochondria–membrane, mitochondria–soluble, nucleus–chromatin, nucleus–non-chromatin, nucleolus, 40S ribosome, 60S ribosome, cytosol, 19S proteasome, 20S proteasome and unassigned. This level of detail shows the strength of this technique in identifying proteins from different organelles and subcellular compartments. In total, the authors were able to identify and quantify 3832 proteins of which they assigned 2634 proteins to distinct subcellular niches mentioned above. The study also addresses issues related to the evolution of apicomplexa, allowing the identification of evolutionary aspects of the different compartments in comparison with other species. Among them, dense granules stand out as the most recent organelles. In other cases, accelerated evolution is observed, such as IMC or nuclear-chromatin cluster proteins, the latter potentially having an impact on the machinery for regulating gene expression. Most rapidly changing proteins are enriched in rhoptries, dense granules, and at the parasite surface, which could be associated with host–parasite coevolution.

## 6. Proteomics Approach in Drug Discovery Efforts

Current drugs for toxoplasmosis primarily target acute infection but are less effective against chronic toxoplasmosis. Identifying new drug targets is crucial to develop improved treatment options that can overcome the limitations of existing drugs. These targets should ideally address both acute and chronic stages of the infection, particularly within the brain and skeletal muscles [171].

Proteomics has provided crucial understandings of *T. gondii*’s biology, identifying potential drug targets, repurposing certain drugs, and contributing to the development of innovative therapeutics, diagnostics, and vaccines against this parasitic pathogen [6,67,171,172,173]. The identification of functional proteins within structures like micronemes and rhoptries holds promise for advancing diagnostics and vaccine development against toxoplasmosis [67,174]. Initially, drugs were discovered through whole organism screening, which involved testing compounds in in vitro and in vivo models. However, recent advances in genomics and proteomics have shifted the focus towards target-based drug design [175]. Integrating multi-omics data, including proteomics, genomics, and transcriptomics, is crucial for understanding parasite biology and identifying new drug and vaccine targets. Effective data management strategies are essential to harness the potential of “big data” in parasitology [176].

### 6.1. Drug Target Identification and Therapeutics

Currently, there are only a few drugs available to treat *T. gondii* infections and some of them have limitations such as toxicity and the potential for parasite resistance. By comparing protein expression profiles between different stages of the parasite’s life cycle and between parasite and host cells, researchers can pinpoint proteins that are specific to the parasite, making them potential targets for drug development. Proteomics has identified key drug targets, such as histone variant H2B.Z acetylation, essential for *T. gondii*’s fitness [39]. Innovative methods like CRISPR-based oligo recombineering (CORe) have been employed to identify chemically reactive sites for drug targeting [177]. Protein phosphatases are promising drug targets against apicomplexan parasites [178]. N-myristoyl transferase (NMT) is considered a potential target [179], and the spider peptide XYP1 demonstrates anti-*T. gondii* effects by interacting with membrane-associated proteins [180]. Identification of unique membrane proteins in *T. gondii* suggests potential candidates for therapeutic development [181].

### 6.2. Insights into Protein Modification and Pathway

Understanding the complex interaction between the parasite and the host is crucial for drug development, as the host’s immune response can influence the efficacy of treatment. PTMs, such as crotonylation and 2-hydroxyisobutyrylation, affect critical enzymes in energy-related pathways. Histone modifications like acetylation play essential roles in gene regulation, and parasite fitness [39]. It is not limited to histone proteins but also extends to nonhistone proteins involved in diverse cellular functions. The presence of acetylation as a regulatory mechanism in protozoan parasites offers an opportunity to explore this machinery as a target for drug development [182]. Disrupting specific protein pathways, such as plastidic iron–sulfur cluster biogenesis, affects parasite viability and cellular functions [183]. It was found that blocking palmitoylation enhances the release of invasion-related proteins, including AMA1, from apical secretory organelles. This observation suggests that AMA1 plays a role in controlling the secretion process of these proteins [111]. That information is crucial for designing drugs that disrupt key pathways or processes in the parasite’s biology while minimizing harm to the host.

### 6.3. Advancements in Diagnostics and Vaccines

Proteomics contributes to diagnostic tests and vaccine development against *T. gondii*. Multiepitope antigens and computational pipelines enhance diagnostic and vaccine strategies. Identifying immunodominant epitopes and functional proteins through proteomics aids in developing comprehensive protection strategies against toxoplasmosis [67]. A significant homology was observed in the antigenic proteome profiles between *N. caninum* and *T. gondii,* which has important implications. It suggests the feasibility of designing multicomponent vaccines that target common antigens shared by both parasites [184]. Proteomics can also play a role in vaccine development by identifying potential protein antigens that can stimulate an immune response [185].

### 6.4. Screening and Testing Drug Candidates

Discovering new drug candidates with anti-toxoplasma activity is a significant challenge. Proteomics facilitates the screening of potential drug compounds by assessing their impact on the parasite’s proteome. Researchers can examine how candidate drugs alter protein expression patterns, helping to identify compounds that effectively inhibit the parasite’s growth or disrupt essential pathways. The investigation of essential proteins of *T. gondii* as potential drug targets has been ongoing for the past two decades. While these efforts have shown promise in vitro, translating these findings into effective treatments for toxoplasmosis in humans has been challenging. The utilization of proteomics-based investigations to examine the interactions between drug candidates and proteins from both the parasite and the host can serve as an effective approach for characterizing drug targets [173]. In another study, in silico screening of compounds was conducted based on the proteome of *T. gondii*. They selected thirteen compounds and evaluated them in vitro against the parasite using a cell-based assay. This approach identified several compounds, including almitrine, bortezomib, and fludarabine, with in vitro anti-*T. gondii* activity. Almitrine, in particular, demonstrated a high selectivity index and showed interactions with specific transporters, suggesting a potential mechanism of action [186].

### 6.5. Characterizing Drug Resistance

*T. gondii* has the potential to develop resistance to existing drugs, reducing their effectiveness. The presence of drug-resistant *T. gondii* strains poses challenges to treatment efficacy, especially in immunocompromised patients. This necessitates a better understanding of resistance mechanisms and the implementation of monitoring programs to address this public health issue [187]. Proteomics can shed light on mechanisms of drug resistance in *T. gondii*. By comparing protein and PTM profiles of drug-sensitive and drug-resistant strains, researchers can identify changes in protein expression that contribute to resistance, informing the design of new drugs or treatment strategies. Proteomic analysis revealed the down- or up-regulation of various proteins, with a particular focus on some key proteins, both actin and MIC8, that play important roles in invasion capability [188]. Doliwa et al. provided valuable proteomic insights into sulfadiazine resistance in *T. gondii* strains isolated from clinical cases, contributing to our understanding of the mechanisms underlying drug resistance in this parasite [29]. Drug resistance in *T. gondii* can also develop when parasite strains are exposed to increasing concentrations of antiparasitic drugs, such as artemisone and artemiside. The development of resistance is associated with alterations in the parasite’s proteome, leading to changes in protein expression, particularly those involved in reactive oxygen species (ROS) regulation. This resistance mechanism differs from that observed in other parasites like *Plasmodium*, highlighting the complexity of drug resistance in *T. gondii* [189].

In summary, recent advances in high-throughput proteomics techniques show potential for identifying parasite-specific markers, especially very low abundance proteins and proteins that have small differences in protein structure but have significant functional consequences in increasing drug specificity and reducing toxicity. Utilizing high-throughput proteomics techniques can significantly reduce the expenses associated with screening and validating drug targets. This cost reduction is particularly advantageous for neglected diseases with limited market potential, such as toxoplasmosis. With the applications of these cutting-edge proteomic technologies in *T. gondii* research, the resolution of these challenges in drug development appears to be on the horizon.

## 7. Future Perspectives

The future of proteomics lies in the accurate and precise measurement of thousands of proteins across thousands of samples. Ongoing progress in proteomic techniques, data analysis, and sample preparation offers exciting prospects for enhancing our understanding of intricate biological systems. Over the past few decades, researchers have encountered challenges in discovering biomarkers within plasma due to the broad dynamic range of proteins. The introduction of groundbreaking MS technologies and innovative sample preparation methods, as discussed earlier, is poised to facilitate the discovery of new biomarkers and enable precision medicine at scale. In recent research, the control of protein translation and degradation has become increasingly important in understanding how organisms quickly adjust to environmental changes. Exploring whether proteins are responsive to different stresses or associated with the invasion and establishment of intracellular parasites undergoing diverse turnover rates would be highly interesting. The use of comparative proteomics, combined with bioinformatics analyses, has the potential to reveal new groups of proteins related to turnover, potentially identifying novel players in each specific situation.

Typically, the implementation of the latest proteomics technologies in *T. gondii* research lags 1–2 years behind their adoption in high-profile human diseases and cancer proteome projects. We anticipate the integration of emerging technologies, including single-cell proteomics, DIA, targeted proteomics, and top-down proteomics, into *T. gondii* research. This expectation is particularly fueled by advancements in mass spectrometers, such as the Orbitrap/Astral dual analyzer and timsTOF Ultra, which hold promise for enhancing the capabilities of proteomic studies in the context of *T. gondii*.

Furthermore, as 90% of the human proteome is mapped [190], future proteomics research, including *T. gondii* studies, will emphasize integrating proteomics with other multi-omics approaches. Artificial intelligence and machine learning will play a critical role in the analysis and interpretation of “big data”. These advancements not only hold promise for identifying novel biomarkers but also offer valuable insights into the invasion mechanisms of *T. gondii* and potential therapeutic targets.

## Data Availability

All of the raw data generated are available upon reasonable request to the corresponding authors.

## Supplementary Materials

The following supporting information can be downloaded at: https://www.mdpi.com/article/10.3390/pathogens13010033/s1, Table S1: A summary of the proteomics applications in *T. gondii*.
